# The NightLife study — the clinical and cost-effectiveness of thrice-weekly, extended, in-centre nocturnal haemodialysis versus daytime haemodialysis using a mixed methods approach: study protocol for a randomised controlled trial

**DOI:** 10.1186/s13063-023-07565-w

**Published:** 2023-08-12

**Authors:** Katherine L. Hull, Kate Bramham, Cassandra L. Brookes, Victoria Cluley, Carmel Conefrey, Nicola J. Cooper, Helen Eborall, James Fotheringham, Matthew P. M. Graham-Brown, Laura J. Gray, Patrick B. Mark, Sandip Mitra, Gavin J. Murphy, Niamh Quann, Leila Rooshenas, Madeleine Warren, James O. Burton

**Affiliations:** 1 Department of Cardiovascular Sciences, University of Leicester, Leicester, UK; 2 John Walls Renal Unit, University Hospitals of Leicester NHS Trust, Leicester, UK; 3 King’s Kidney Care, King’s College Hospital, London, UK; 4 Department of Women and Children’s Health, Faculty of Life Sciences and Medicine, King’s College London, London, UK; 5 Leicester Clinical Trials Unit, University of Leicester, Leicester, UK; 6 School of Sociology and Social Policy, University of Nottingham, Nottingham, UK; 7 Bristol Population Health Science Institute, University of Bristol Medical School, Bristol, UK; 8 Department of Population Health Sciences, University of Leicester, Leicester, UK; 9 College of Medicine and Veterinary Medicine, Usher Institute, University of Edinburgh, Edinburgh, UK; 10 Health Economics and Decision Science, School of Health and Related Research, University of Sheffield, Sheffield, UK; 11 Sheffield Kidney Institute, Sheffield Teaching Hospitals NHS Foundation Trust, Sheffield, UK; 12 Institute of Cardiovascular and Medical Sciences, University of Glasgow, Glasgow, UK; 13 Manchester Institute of Nephrology and Transplantation, Manchester Academic Health Science Centre, Research and Innovation, Manchester University NHS Foundation Trust, Manchester, UK; 14 Cardiovascular Research Centre, University of Leicester, Leicester, UK; 15 Warren-Charnock Associates, London, UK

**Keywords:** In-centre nocturnal haemodialysis, End-stage kidney disease, Quality of life, Cost-effectiveness

## Abstract

**Background:**

In-centre nocturnal haemodialysis (INHD) offers extended-hours haemodialysis, 6 to 8 h thrice-weekly overnight, with the support of dialysis specialist nurses. There is increasing observational data demonstrating potential benefits of INHD on health-related quality of life (HRQoL). There is a lack of randomised controlled trial (RCT) data to confirm these benefits and assess safety.

**Methods:**

The NightLife study is a pragmatic, two-arm, multicentre RCT comparing the impact of 6 months INHD to conventional haemodialysis (thrice-weekly daytime in-centre haemodialysis, 3.5–5 h per session). The primary outcome is the total score from the Kidney Disease Quality of Life tool at 6 months. Secondary outcomes include sleep and cognitive function, measures of safety, adherence to dialysis and impact on clinical parameters. There is an embedded Process Evaluation to assess implementation, health economic modelling and a QuinteT Recruitment Intervention to understand factors that influence recruitment and retention. Adults (≥ 18 years old) who have been established on haemodialysis for > 3 months are eligible to participate.

**Discussion:**

There are 68,000 adults in the UK that need kidney replacement therapy (KRT), with in-centre haemodialysis the treatment modality for over a third of cases. HRQoL is an independent predictor of hospitalisation and mortality in individuals on maintenance dialysis. Haemodialysis is associated with poor HRQoL in comparison to the general population. INHD has the potential to improve HRQoL. Vigorous RCT evidence of effectiveness is lacking. The NightLife study is an essential step in the understanding of dialysis therapies and will guide patient-centred decisions regarding KRT in the future.

**Trial registration:**

Trial registration number: ISRCTN87042063. Registered: 14/07/2020.

## Administrative information

**Table:** 

Title {1}	The NightLife study—the clinical and cost effectiveness of thrice weekly, extended, in-centre nocturnal haemodialysis versus daytime haemodialysis using a mixed methods approach: study protocol for a randomised controlled trial
Trial Registration {2a and 2b}	Trial registration number: ISRCTN87042063 Prospectively registered: 14/07/2020
Protocol version {3}	Version 4, 09/02/2023
Funding {4}	The NightLife study is funded by the National Institute for Health Research (NIHR) Health Technology Assessment (HTA) award [HTA (NIHR127440)]
Author details {5a}	Department of Cardiovascular Sciences, University of Leicester; John Walls Renal Unit, University Hospitals of Leicester NHS Trust; King’s Kidney Care, King’s College Hospital; Department of Women and Children’s Health, Faculty of Life Sciences and Medicine, King’s College London; Leicester Clinical Trials Unit, University of Leicester; School of Sociology and Social Policy, University of Nottingham; Bristol Population Health Science Institute, University of Bristol Medical School; Department of Population Health Sciences, University of Leicester; Usher Institute, College of Medicine and Veterinary Medicine, University of Edinburgh; Health Economics and Decision Science, School of Health and Related Research, University of Sheffield; Sheffield Kidney Institute, Sheffield Teaching Hospitals NHS Foundation Trust; Institute of Cardiovascular and Medical Sciences, University of Glasgow; Manchester Institute of Nephrology and Transplantation; Manchester Academic Health Science Centre, Research and Innovation, Manchester University NHS Foundation Trust; Cardiovascular Research Centre, University of Leicester; Warren-Charnock Associates; NightLife study group – see acknowledgments
Name and contact information for the trial sponsor {5b}	Research Governance Office Research & Enterprise Division University of Leicester University Road Leicester LE1 7RH UK rgosponsor@leicester.ac.uk
Role of sponsor {5c}	The Sponsor will be responsible for overall oversight of the study. The Sponsor will delegate duties to other parties, including Leicester Clinical Trials Unit, this delegation will be formally documented. The Sponsor will not be part of the study conduct, data analysis and interpretation, manuscript writing, and dissemination of results

## Introduction

### Background and rationale {6a}

Nearly 70,000 adults in the UK receive kidney replacement therapy (KRT) for end-stage kidney disease (ESKD) [1]. Haemodialysis accounts for over a third of KRT treatment in the UK [1] and 89% of all dialysis worldwide [2]. Despite its value as a life-saving treatment in individuals unable to receive or awaiting kidney transplantation, individuals receiving maintenance haemodialysis have poorer health-related quality of life (HRQoL) than the non-dialysis population, which associates significantly with adverse outcomes, such as hospitalisation and mortality [3–5], and increased co-morbidity, including frailty, reduced physical function and depression [5, 6].

The reasons for poor HRQoL amongst the haemodialysis population are multifactorial and reflect the complex interplay between the constructs of the biopsychosocial model of health [7]. For instance, individuals with kidney failure experience substantial symptom burden, must adjust and cope with living with a challenging chronic illness and are restricted in their time, travel and work [8–10]. Extrapolate this to a societal level, kidney failure and haemodialysis exerts significant strain on the health economy [11].

The individual burden of living with ESKD is compounded by dialysis treatment regimens. The majority of haemodialysis care takes place ‘in-centre’ at a hospital-based or satellite haemodialysis unit [1]. In-centre haemodialysis treatment regimens are rigid; patients must attend thrice-weekly (typically either Monday, Wednesday and Friday, or Tuesday, Thursday and Saturday) with treatment times limited to 3.5 to 5 h per session. This is not at the convenience for the individual, but rather suits the service capacity requirements and managing patient numbers, whilst providing an adequate prescription of dialysis [12].

Individuals receiving haemodialysis within their home experience better quality of life and long-term clinical outcomes in comparison to in-centre haemodialysis [13, 14]. Home haemodialysis offers individuals flexibility regarding the length of dialysis sessions, time of day that treatment takes place and frequency of their treatment. Consequently, individuals can dialyse for longer, mimicking native kidney function to a greater extent compared to in-centre regimens, but with greater autonomy over the schedule. However, home haemodialysis is only utilised by approximately 2% of the UK KRT population [1]; there are considerable professional, patient and societal-related barriers to expanding home haemodialysis services [15, 16]. Alternative haemodialysis regimens are needed to address the dichotomy between in-centre and home haemodialysis therapies.

In-centre nocturnal haemodialysis (INHD) is an alternative regimen whereby individuals have the opportunity to dialyse overnight at a hospital-based or satellite dialysis unit, for 6 to 8 h per session. This increases the total weekly dialysis time to 18 to 24 h, a substantial increase compared to the typical 12 h of daytime in-centre haemodialysis, without impinging on dialysis-free time during the day. There is a growing body of evidence from observational data demonstrating that INHD is associated with improvements in HRQoL [17–20], dialysis adequacy [20–22], surrogates for cardiovascular health [23], survival [21, 24] and clinical parameters (e.g. anaemia and markers of renal bone disease) [17, 21–25]. It is probable that the reason for these observed benefits are due to both the increased dialysis dose and the overnight scheduling, which essentially increases the amount of usable dialysis-free time in comparison to usual in-centre daytime haemodialysis [26]. The extrapolation of these observational findings to the haemodialysis population are limited by significant sources of bias, in particular, selection bias and lack of randomisation, short follow-up period, small sample sizes and inadequate study power.

There have been three key randomised clinical trials (RCTs) evaluating the impact of extended-hours nocturnal haemodialysis in comparison to standard care (in-centre daytime haemodialysis) on HRQoL: the Alberta trial [27], the Frequent Haemodialysis Network (FHN) Nocturnal Trial [28] and the ACTIVE dialysis trial [29]. Disappointingly, all three RCTs did not show a statistically significant improvement in HRQoL with extended-hours nocturnal haemodialysis. Furthermore, the FHN Nocturnal trial demonstrated that allocation to the intervention hastened residual kidney function decline in comparison to the control [30] and a trend towards increased vascular access events with extended-hours nocturnal haemodialysis [28], although this did not reach statistical significance and synthesised data from all three trials indicated there was no difference in vascular access events between intervention and control [31].

These findings need to be interpreted with caution when considering their relevance to INHD. All three RCTs provided a haemodialysis prescription which may be considered *intensive* (i.e. increased duration *and* frequency of haemodialysis) rather than just *extended:* the ACTIVE dialysis trial aimed to achieve ≥ 24 h of haemodialysis a week across a minimum of three dialysis sessions [29]; the FHN Nocturnal Trial intervention involved ≥ 6 h of haemodialysis six times a week [28]; and the Alberta trial intervention required ≥ 6 h of haemodialysis five to six times a week [27]. In all three RCTs, the majority of the nocturnal haemodialysis was performed at home; when only 2% of the UK KRT population utilise home haemodialysis, the applicability of the findings to the usual haemodialysis population is limited.

There is an absence of RCT data dedicated to evaluating the potential benefits and safety of thrice-weekly extended-hours INHD in a UK population. Without robust evidence, expansion of alternative in-centre haemodialysis regimens is restricted. This will be addressed by the NightLife study, a pragmatic multicentre RCT to evaluate the impact of INHD on HRQoL, clinical and cost-effectiveness, and safety in comparison to standard in-centre daytime haemodialysis.

### Aim and objectives {7}

The overall aim of the NightLife study is to test the clinical and cost-effectiveness of 6 months of thrice-weekly, extended-hours (6 to 8 h) INHD compared to standard care haemodialysis (thrice-weekly, 3.5 to 5 h of in-centre haemodialysis during the day) for adults requiring maintenance haemodialysis. This will be achieved with the following objectives:

1. To determine the willingness and ability to recruit and randomise individuals to thrice-weekly, extended-hours INHD.
2. To measure the effect of 6 months of extended-hours INHD compared with daytime haemodialysis on quality of life.
3. To measure the effect of 6 months of extended-hours INHD compared with daytime haemodialysis on sleep, fatigue, dialysis recovery, cognitive function, clinical parameters and dialysis adequacy and safety (including residual kidney function and vascular access complications).
4. To assess feasibility and acceptability of extended-hours INHD to individuals receiving long-term in-centre haemodialysis, their family and care givers, and staff, in order to identify contextual factors that influence the implementation of INHD.
5. To evaluate the cost-effectiveness of 6 months of extended-hours INHD compared with daytime haemodialysis from a health and social care perspective.
6. To understand and address issues that undermine recruitment and retention in the NightLife study.

### Trial design {8}

**Fig. 1:**
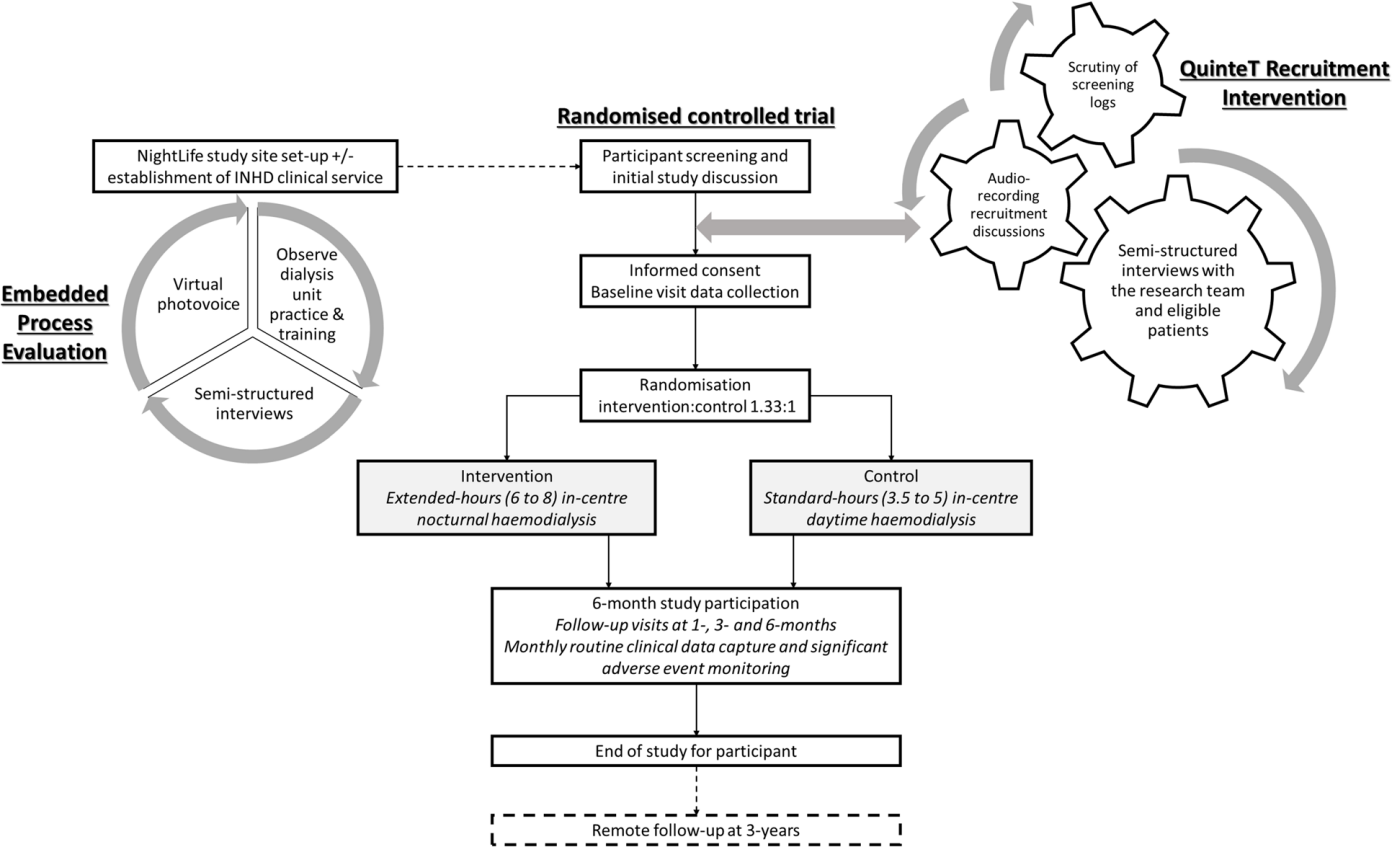
Overview of the NightLife study: the main randomised controlled trial, embedded Process Evaluation to provide a formative evaluation of study processed and intervention implementation, and the QuinteT Recruitment Intervention to optimise recruitment practices and maintain clinical equipoise in study discussions

NightLife is a superiority study delivered as a two-arm, parallel, pragmatic, multicentre RCT. There is an embedded process evaluation, to assess the study’s processes and intervention implementation, and cost-effectiveness analysis. An integrated QuinteT recruitment intervention (QRI) will study attitudes towards recruitment and equipoise [32]. An overview of the NightLife study is demonstrated in Fig. 1.

## Methods: participants, interventions and outcomes

### Study setting {9}

The NightLife study will take place across approximately 18 participating dialysis units, from within the NHS and commercial providers across the UK. Participating dialysis units are eligible from any type of NHS Trust and in any location across the UK providing they have a research ethics department and can deliver the required research activities (i.e. recruitment and data collection) and clinical service provision (i.e. provide an existing or new INHD service). The list of participating sites is available on the NightLife study clinical trials registration page (ISRCTN87042063).

### Eligibility criteria {10}

The eligibility criteria for the main RCT, embedded process evaluation and QRI are reported in Table 1.

**Table 1:** Eligibility criteria for the NightLife study

**Workstream 1 (main randomised controlled trial)**
Inclusion criteria:
• Adults (≥ 18 years);
• End-stage kidney failure receiving thrice-weekly in-centre haemodialysis for at least 3 months
• Able to provide written informed consent;
• Able participate fully in follow-up
Exclusion criteria:
• Currently receiving in-centre nocturnal haemodialysis;
• < 3 months since stopping extended daytime dialysis or in-centre nocturnal haemodialysis;
• Extended-hours haemodialysis is clinically indicated (e.g. calciphylaxis, pregnancy)
• Scheduled living-donor transplant in the next 6 months;
• Plans to change dialysis modality in the next 6 months;
• Life expectancy < 6 months;
• Current participation in an interventional trial with conflicting therapies or primary outcome
**Workstream 2 (Process Evaluation)**
Staff inclusion criteria
• Clinical and non-clinical staff working at haemodialysis units participating in the NightLife study;
• Adults (≥ 18 years);
• Ability to give written informed consent;
• Ability and willingness to participate fully in the observations and interviews
Relative/visitor inclusion criteria
• Relatives or visitors accompanying patients (that fulfil workstream 1 eligibility criteria) to haemodialysis sessions;
• Adults (age ≥ 18 years);
• Ability to give written informed consent;
• Ability and willingness to participate fully in the observations and interviews
**Workstream 3 (QuinteT recruitment intervention)**
Staff inclusion criteria
• Clinical and non-clinical staff working involved in overseeing or recruiting to the NightLife study;
• Adults (≥ 18 years);
• Ability to give informed consent

### Who will take informed consent? {26a}

Informed consent will be received by members of the research team (research nurses and clinicians) trained in Good Clinical Practice. Recruitment and informed consent will take place on the dialysis units during potential participants’ usual haemodialysis sessions.

### Additional consent provisions for collection and use of participant data and biological specimens {26b}

Informed consent includes access to medical records and linkage of research data to routine health data relevant to study follow-up. This trial does not involve collecting biological specimens for storage. Separate informed consent is required for the main RCT, embedded process evaluation and integrated QRI. Participation to the main RCT is not required for an individual to join the process evaluation or QRI.

There is an optional cardiac magnetic resonance imaging sub-study for participants joining the main RCT, for which additional informed consent is required. This sub-study protocol will be published separately and is not detailed in this manuscript.

### Explanation for the choice of comparators {6b}

The control group is the typical haemodialysis regimen prescribed in routine nephrology clinical practice: in-centre day time haemodialysis. The intervention is the alternative regimen of thrice-weekly extended-hours INHD, which currently is not widely available within the NHS but has been associated with a number of potential benefits.

### Intervention description {11a}

Participants allocated to the intervention group will receive thrice-weekly, extended-hours (6 to 8 h) INHD for 6 months. The participants allocated to the control arm will continue with thrice-weekly, standard-hour (3.5 to 5 h) in-centre daytime haemodialysis. All other aspects of dialysis care will remain the same.

### Criteria for discontinuing or modifying allocated interventions {11b}

Participants may withdraw from complying with the allocated study treatment arm and/or providing data to the study, at any time, for any reason without affecting their usual care. Should a participant wish to withdraw from receiving their allocated study treatment, efforts will be made to continue to obtain follow-up data, with their permission. Participants do not have to give a reason for withdrawal; however, if they do provide a reason for leaving the study, this will be recorded.

The clinical team and/or investigators may discontinue a participant from the study at any time if considered necessary, e.g. based on symptoms and blood test results.

### Strategies to improve adherence to interventions {11c}

The integrated QRI aims to enhance retention to the NightLife study, as outlined in ‘Recruitment {15}’ below.

### Relevant concomitant care permitted or prohibited during the trial {11d}

All other aspects of dialysis care will remain the same during participation in the NightLife study.

### Provisions for post-trial care {30}

At the end of the 6-month study period, participants allocated to the control arm will have the option of switching to INHD as per capacity of local clinical service. Similarly, participants allocated to INHD have the option to remain on this haemodialysis regimen or return to usual daytime haemodialysis.

### Outcomes {12}

#### Primary outcome measure

The primary outcome is the Kidney Disease Quality of Life (KDQoL) total score measured over 6 months. The KDQoL is a kidney disease-specific measure of HRQoL and has been shown to have reliability and construct validity amongst dialysis patients [33]. The KDQoL is available in different formats; the KDQoL Short Form (KDQoL-SF) questionnaire is being used for the NightLife study. The KDQoL-SF questionnaire includes the generic Short Form-36 as its core with an additional 43 kidney disease-targeted items. The primary outcome (KDQoL total score) is calculated from a subset of data collected in the KDQoL-SF questionnaire.

The KDQoL incorporates four domains: physical component summary score (PCS), mental component summary score (MCS), kidney summary score (KSS) and the kidney disease component summary score (KDCS). The results for each of the dimensions are all generated from the data collected in the KDQoL-SF questionnaire. As well as being a sensitive measure of quality of life, each 1-point improvement in the PCS is associated with reductions in both the relative risk of death and hospitalisation by 2% [34]. Similarly, a 1-point improvement in MCS has been associated with a relative risk reduction for death of 2% and for hospitalisation by 1% [34]. Given that there is an almost linear relationship between lower score and increased hospitalisation and mortality [35], a 5-point difference over 6 months in KDQoL physical score would associate with a clinically significant 5–10% reduction. This 5-point difference equates to a standardised difference of 0.26. The domains of the KDQoL-SF, alongside hospitalisation and mortality, have all been highlighted by the Standardised Outcomes in Nephrology (SONG-HD) initiative as priority outcomes [36]; thus, the KDQoL measures what is most important to individuals requiring maintenance haemodialysis.

#### Secondary outcome measures

The secondary outcome measures will further explore quality of life; evaluate sleep, dialysis recovery and cognitive function; assess safety of the intervention and impact on clinical parameters; and determine adherence to the intervention. The secondary outcome measures, assessment tools and follow-up time points (after baseline data collection) are reported in Table 2.

**Table 2:** Secondary outcome measures for Workstream 1 (main randomised controlled trial)

Secondary outcome measures	Follow-up
**Patient reported outcome measures**	
KDQoL total score	1, 3 and 6 months
KDQoL domains: Physical Component Summary Score (PCS), Mental Component Summary Score (MCS), Kidney Summary Score (KSS), Kidney Disease Component Summary Score (KDCS)	6 months
EuroQol EQ-5D-5L: will be used to determine health state descriptions for the five components (mobility, self-care, usual activities, pain/discomfort, anxiety/depression) combined with health-related quality of life index scores to generate quality-adjusted life year (QALY) profiles for the cost-effectiveness analysis	1, 3 and 6 months
The SONG-HD fatigue score to evaluate fatigue experience by individuals on long-term dialysis	1, 3 and 6 months
Pittsburgh Sleep Quality Index (PSQI); a validated tool to assess sleep quality in people on dialysis and the association between sleep and lower health-related quality of life	1, 3 and 6 months
Time to recover in minutes after dialysis; a simple, reliable and sensitive validated tool	1, 3 and 6 months
**Cognitive function**	
The Montreal Cognitive Assessment (MoCA) will be used to explore changes in cognitive function. The MoCA is a well-known validated tool for assessing cognitive health and can be used for individuals on long-term dialysis	3 and 6 months
**Measures of safety**	
44-h intradialytic urine collection with paired blood samples to estimate residual kidney function	6 months
Serum beta-2 microglobulin, a validated surrogate for residual kidney function in individuals on long-term haemodialysis	Monthly
Serious adverse events (SAEs):	Monthly
• SAEs in totality (rate/years)	
• Vascular access complications that lead to SAEs (rate/years)	
• Dialysis prescription changes that lead to SAEs (rate/years)	
Clinical events:	Monthly
• Cardiovascular events (rate/years)	
• Cardiovascular mortality (rate/years)	
• Overall mortality (rate/years)	
**Impact on clinical parameters**	
Blood results: haemoglobin, ferritin, transferrin saturation, calcium, potassium, phosphate, parathyroid hormone	Monthly
Dialysis adequacy assessed via urea reduction ratio and Kt/V (determined from pre-dialysis urea, post-dialysis urea, post-dialysis weight and ultrafiltration volume	Monthly
Pre-dialysis blood pressure	Monthly
Medication prescription: antihypertensive agents, phosphate binders, potassium binders, erythropoietin, iron supplementation	6 months
**Adherence to allocated study arm**	
Number of missed dialysis sessions	Monthly
Minutes per dialysis session	Monthly
Number of dialysis sessions not achieving time criteria	Monthly
Number of temporary changes from treatment allocation (i.e. participant allocated to intervention dialysing during the day	Monthly
**Cost-effectiveness**	
Resource use and expenditure questionnaire	1, 3 and 6 months

### Participant timeline {13}

Overview of participant timeline and schedule of events is summarised in Fig. 1. The schedule of enrolment, randomisation, assessments and follow-up are summarised in Table 3.

**Table 3:** The schedule of enrolment, randomisation, assessments and follow-up

Procedure	Screening	Baseline	1 month	2 months	3 months	4 months	5 months	6 months	3 years
Review eligibility criteria	X	X							
Written informed consent		X							
Demographic data and medical history		X							
Questionnaires: KDQoL, EQ-5D-5L, PSQI, SONG-HD, TTR		X	X		X			X	
MoCA		X			X			X	
Baseline health economics questionnaire		X							
Randomisation		X							
Intradialytic urine collection and paired blood samples*		X						X	
Serum beta-2 microglobulin*		X	X	X	X	X	X	X	
Resource use and expenditure questionnaire			X		X			X	
Routine clinical data^◊^ including adherence, dialysis adequacy, blood pressure and monthly blood results		X	X	X	X	X	X	X	
Capture of routine clinical data and resource usage (UKRR, SRR, HES, ONS, ISD)									X

*Blood samples drawn at the same time as routine clinical testing. ^◊^Routinely collected data on all dialysis units

### Sample size {14}

The recruitment target is 350 participants; 150 to the control arm and 200 to the intervention arm. This recruitment target consists of both the sample size required to achieve study power and considerations for participant drop-out.

The study is powered to detect a standardised difference of 0.26 in the KDQoL total score between groups over 6 months, adjusting for baseline KDQoL total score with correlation coefficient of 0.78. To achieve 90% power and a type I error rate of 5%, 252 participants are required. Assuming an overall attrition rate of 15%, and 25% non-adherence with INHD in the intervention group, the targeted number of randomisations is 350 (150 to control arm, 200 to intervention arm). This will ensure adequate power for both the intention to treat and per-protocol analyses.

Recruitment will be reviewed by the independent Data Safety and Monitoring Committee to ensure the study remains adequately powered with respect to participant retention and permanent treatment arm crossover. Furthermore, as additional publications reporting the KDQoL-36 short form become available [37], the assumptions regarding the correlation coefficient between measurements during follow-up will be reviewed to ensure maximal study power is achieved.

### Recruitment {15}

The inclusion criteria for the NightLife study are broad, with participation to the main RCT accessible to the majority of adults receiving maintenance in-centre haemodialysis. Initially, patients at participating dialysis units will be ‘pre-screened’ to identify adults receiving maintenance haemodialysis (≥ 3 months of haemodialysis) who do not have a planned living-related kidney transplantation through liaison with the clinical teams. As randomisation to the intervention arm relies on the availability of an INHD appointment time within usual NHS service deliver, once an INHD appointment becomes available, pre-screened patients will be approached during routine haemodialysis sessions and formerly screened to confirm eligibility and gauge interest in the NightLife study. Patients interested in the study will be provided with a patient information sheet and be given a minimal of 48 h (i.e. 2 days between routine haemodialysis sessions) before they are revisited for re-discussion. At this second visit, the NightLife study discussion will either result in informed consent, study participation declined or study discussions paused (e.g. participant may require more time to consider their decision, or they would like to delay joining the study to a point that is more convenient around lifestyle factors such as work). The stages of pre-screening, screening and recruitment will be conducted by members of the research team (clinicians and research nurses) with Good Clinical Practice training.

The availability of INHD appointment slots is key to the ability of the research team to approach, consent and randomise participants. Consequently, recruitment rates will vary across sites according to their INHD status: *established* sites will already have an INHD service available and access to the study will compete with existing routine clinical care; *naïve* sites will set up an INHD service through the study and so the majority of the appointment times for INHD will be utilised by NightLife study participants. This creates two recruitment trajectories: one to two recruits per month at *established* INHD sites as appointments become available; and an initial large recruitment of participants (9 to 15) at *naïve* INHD sites as participants are randomised to either fill the INHD appointment times or remain on their usual haemodialysis. With only three participating sites providing an established INHD service, recruitment to the NightLife study will occur in stepwise manner as sites join. The NightLife study opened to recruitment in October 2021 and will close to recruitment in March 2024.

Recruitment and retention to NightLife was anticipated to be potentially challenging due to concerns around healthcare professionals’ and potential participants’ perceptions of equipoise. In particular, patient and public contributors expressed concern that patients may be disappointed and experience resentful demoralisation if not allocated to receive INHD. Resentful demoralisation can negatively impact study continuation and bias the results; participants allocated to the control arm may be more likely to drop-out or crossover, be more attentive to adverse event reporting and express lower quality of life [38–40]. This underscored the importance of conveying the rationale for NightLife and the potential benefits and disadvantages of the two treatment arms when presenting the RCT to potential participants [41]. An integrated QRI was incorporated into the NightLife protocol to understand and address factors that undermine recruitment and retention to the RCT. It comprises:

- Pre-emptive strategies to support recruitment, delivered prior to sites opening (e.g. training to raise awareness of clinical equipoise; strategies for explaining NightLife to potential participants with a view to safeguarding informed consent; input with preparation of patient-facing trial documentation, and designing tools to monitor recruitment, e.g. screening logs).
- Mixed methods to investigate recruitment and retention issues as they arise in NightLife, including (i) quantitative analyses of screening log data; (ii) semi-structured interviews with site personnel; (iii) audio-recordings of site personnel presenting the NightLife study to potential participants; and (iv) interviews with patients who accept and decline participation in the RCT. Data will be triangulated to crystallise key explanations for recruitment/retention issues [42].
- Delivery of tailored actions to address recruitment and retention issues as the RCT is underway, informed by findings from the above (‘b’) and discussion with the Trial Management Group.

Investigation of recruitment issues and delivery of tailored actions will proceed cyclically throughout the NightLife recruitment period.

### Assignment of interventions: allocation

#### Sequence generation {16a}

Participants will be randomised after informed consent has been provided and baseline questionnaire data collected. Allocation will be weighted towards to the intervention (1:1.33, control:intervention) to account for the assumed 25% conversion back to in-centre daytime haemodialysis from the intervention group within the first 2 weeks (see above). Randomisation will be stratified by haemodialysis unit and age (< 65 years, ≥ 65 years) and managed by the Leicester Clinical Trials Unit.

#### Concealment mechanism {16b}

Randomisation will be completed via a web-based system (Sealed Envelope) [43] to maintain allocation concealment.

#### Implementation {16c}

Randomisation is completed by the research team using a web-based system (Sealed Envelope) [43] once informed consent has been achieved and baseline questionnaire data have been collected.

### Assignment of interventions: blinding

#### Who will be blinded? {17a}

All analysis will be conducted by an assessor blinded to any patient data or intervention allocation; participants and investigators are not blinded due to the nature of the intervention and control.

#### Procedure for unblinding if needed {17b}

There is no requirement for a procedure to undertake unblinding as it is an open-label study.

### Data collection and management

#### Plans for assessment and collection of outcomes {18a}

Quality of life questionnaire data collection will be conducted by the research delivery teams at participating sites during haemodialysis appointments or over the telephone, at the convenience of the participant, to avoid additional study visits. Clinical information and results will be collected from routine clinical testing and medical records. Data entry will be completed by the researchers at participating sites. The NightLife study dataset will be analysed by the Leicester Clinical Trials Unit.

#### Plans to promote participant retention and complete follow-up {18b}

Study participation on the allocated treatment arm is 6 months. The baseline visit and all follow-up appointments are completed during usual routine haemodialysis appointments. There is the option for quality of life questionnaire data to be completed over the phone if more convenient to the participant. After baseline, the follow-up visits occur at 1, 3 and 6 months. Irrespective of KRT modality after study participation, capture of routine clinical data will take place on the 3-year anniversary of the last participant last visit.

For participants that leave their allocated treatment intervention (i.e. permanent crossovers), permission will be sought to continue to collect follow-up data. Some data may not be available due to death, kidney transplantation and voluntary withdrawal (these losses have been taken into consideration for the sample size calculation) or lack of completion of individual data items. Where possible, the reasons for missing data will be ascertained and reported. Although every effort will be made to minimise crossovers from both treatment arms, the numbers, direction and reasons for participants moving between arms will be recorded and reported in line with CONSORT (Consolidated Standards of Reporting Trials) guidance.

### Data management {19}

A validated web-based Remote Data Capture system provided by the Leicester Clinical Trials Unit will be used for data entry. The NightLife study dataset will be held by the Leicester Clinical Trials Unit. Source data will also be documented on case report forms and stored securely at participating centres in study investigator site files.

### Confidentiality {27}

All participants will be allocated an individual trial identification number. Participants’ personal data included in study-related databases shall be treated in confidence and in compliance with ICH-GCP, the UK Policy Framework for Health and Social Care, and the EU General Data Protection Regulation (GDPR). When processing or archiving personal data, the Sponsor or its representative will take all appropriate measures to safeguard and prevent access to this data by any unauthorised third party.

### Plans for collection, laboratory evaluation and storage of biological specimens for genetic or molecular analysis in this trial/future use {33}

Biological specimens are not being collected for the NightLife study. Blood and urine results for the secondary outcomes are obtained from routine clinical testing, and are all handled in UK NHS laboratories.

## Statistical methods

### Statistical methods for primary and secondary outcomes {20a}

#### Primary outcomes

The primary outcome will compare the KDQoL composite score over 6 months between the intervention and control. The primary outcome analysis will be conducted using a modified intention to treat with participants with at least one post baseline measurement of KDQoL analysed in the groups to which they were randomly allocated, regardless of haemodialysis schedule they actually received. The KDQoL total score over 6 months (i.e. 1, 3 and 6 months) will be compared between the treatment arms using a repeated measures mixed linear regression model with participant as a random effect to account for repeated measures over time. The model will be adjusted for a treatment group, minimisation factors (haemodialysis unit and age) with haemodialysis unit as a random effect and baseline KDQoL total score. Treatment comparison estimates will be presented as adjusted mean difference and 95% confidence intervals.

#### Secondary outcomes

The secondary outcomes will be analysed using linear regression for single point in time continuous outcome measures; repeated measures mixed linear regression model for secondary outcomes measured repeatedly over time; and logistic regression for binary measures. All analyses will be adjusted for baseline value and the minimisation factors (haemodialysis unit and age). The number of deaths and cardiovascular events expected is relatively low; therefore, formal time to event analyses will not be conducted. Kaplan–Meier will be presented to describe the relationship between these events and treatment.

### Interim analyses {21b}

No interim analyses have been pre-defined; formal interim analyses where the treatment groups are compared to evaluate primary or secondary outcome measures are not planned.

### Methods for additional analyses (e.g. subgroup analyses) {20b}

There is increasing demand by clinicians and patients for ‘per-protocol’ analyses which quantify the effect of being randomised to, receiving and continuing a treatment [44]. A secondary, complier average causal effect (CACE) analysis will be conducted to understand the efficacy of extended-hours INHD in those that receive it as planned.

Clinical experience suggests that the development of new conditions which cause instability of the patient whilst they are on haemodialysis may mean that patients allocated to INHD will have to return to usual care during the day. The effect of such switching on the treatment effect found will be assessed.

Subgroup analyses will be limited to the same variables used as minimisation variables. Tests for statistical heterogeneity (e.g. by including treatment group by subgroup interaction parameter in the regression model) will be performed prior to any examination of effect estimate within subgroups.

#### Embedded process evaluation

An embedded process evaluation will provide formative assessment of NightLife study processes and INHD implementation. Data will be collected using ethnographic methods: observations; patient, carer and staff interviews; PhotoVoice [45]; and document collation. This qualitative approach will generate a picture of usual care (in-centre daytime haemodialysis), provide formative feedback on INHD starter packs (advice and resources to facilitate INHD implementation), explore patient and staff views on feasibility and acceptability of INHD, and identify contextual factors influencing its implementation.

#### Embedded health economic evaluation

A health economic evaluation will be completed to assess the cost-effectiveness of INHD in comparison to usual care (in-centre daytime haemodialysis). Healthcare-related resource use, alongside patient- and carer-borne resource use, will be collected and combined with unit costs using standard validated external tools [46–48]. In addition, quality-adjusted life years (QALYs) will be derived from HRQoL data collected from RCT participants. The cost data and QALY data will be combined to estimate the incremental cost per (QALY) gained. The long-term economic impact will be assessed through the development of a probabilistic state transition decision model [49] developed by using observed differences in relevant clinical parameters to extrapolate the health effects of INHD beyond the trial follow-up period, in conjunction with clinical expert input, existing literature and external data sources.

### Methods in analysis to handle protocol non-adherence and any statistical methods to handle missing data {20c}

Adherence to allocated treatment arm is a secondary outcome measure (Table 2), and the data will be analysed as reported for the secondary outcomes.

By design, there will be no missing data for the minimisation factors (haemodialysis unit and age). If the KDQoL outcome is missing for < 5% of the study population over 6 months, a complete case analysis will be conducted. If there is ≥ 5% of participants with a missing post baseline KDQoL score, multiple imputation will be used. Multiple imputation replaces missing values with multiple sets of simulated values to complete the data, performs standard analysis on each completed dataset and adjusts the obtained parameter estimates for missing-data uncertainty using Rubin’s rules to combine estimates.

### Plans to give access to the full protocol, participant-level data and statistical code {31c}

Applications to access anonymised patient-level data can be made to the Trial Management Group (TMG). The full protocol will be available as a supplement upon publication of the primary results paper.

## Oversight and monitoring

### Composition of the coordinating centre and trial steering committee {5d}

The study sponsor is the University of Leicester, and the coordinating centre is the Leicester Clinical Trials Unit. The TMG will meet regularly to review overall study progress and procedure: site set-up, recruitment, completion, safety, protocol implementation and review. An independent Trial Steering Committee will be responsible for the scientific and ethical conduct of the study and will supervise progress of the study.

#### Patient Public Involvement and Engagement (PPIE)

The Leicester PPIE group, consisting of 80 individuals with kidney disease alongside their relatives and carers, first highlighted INHD as an area requiring further research and implementation within clinical service development. This informed the creation of the INHD service across the Leicester Renal Network and generation of pilot data regarding the impact on quality of life and clinical outcomes [20, 26]. The Leicester PPIE group’s ideas and concerns directly informed NightLife study design.

The NightLife study has a PPIE co-applicant to act as a non-independent, non-voting lay representative and expert patient on the Trial Steering Committee. Since NightLife study commencement, a dedicated NightLife study virtual PPIE forum has been established with UK-wide participation. The purpose of this PPIE forum is to ensure the aims and objectives of the study remain focused on issues that matter to patients and the public, highlight known and potential problems in the set-up and delivery of in-centre nocturnal dialysis and ensure that the work is disseminated effectively to the general population. The NightLife PPIE forum convenes biannually (at a minimum) with additional PPIE drop-in sessions in the interim.

### Composition of the data monitoring committee, its role and reporting structure {21a}

An independent Data Safety Monitoring Committee will meet to monitor safety and effectiveness data at least every 12 months; meetings will also be held as necessary should any urgent issues occur.

### Adverse event reporting and harms {22}

The safety of the participants will be monitored for the duration of the 6-month follow-up period. It is anticipated that the study population will experience a significant number of underlying health conditions and consequently an increased number of expected hospital admissions [50, 51]. Adverse events (such as constipation, diarrhoea and headache) will not be collected or reported. Serious adverse events (SAEs) will be collected and are included in the secondary outcomes (Table 2). SAE data will be reported at regular Data Safety and Monitoring Committee meetings; the outcome of this review will guide the Trial Steering Committee and Sponsor on the suitability of study continuation and whether additional data and/or analyses is required.

Expedited reporting of SAEs to the Sponsor will only occur when they are clearly related to the study intervention and of a serious nature. These SAEs include vascular access adverse events (i.e. needle dislodgement during haemodialysis) and events that occur as a direct consequence of haemodialysis prescription, e.g. hypokalaemia, hypophosphataemia.

All serious adverse events will be tabulated and summarised by treatment group, according to system organ class and preferred term, as classified in the Medical Dictionary for Regulatory Activities (MedDRA) [52]. No formal statistical testing will be performed. All events will be summarised by seriousness, expectedness and relatedness.

### Frequency and plans for auditing trial conduct {23}

The Sponsor operates a risk-based monitoring and audit programme, to which this study will be subject. The Leicester Clinical Trials Unit operates a Quality Management System, which will apply to this study with quality checks and quality assurance audits performed as required.

### Plans for communicating important protocol amendments to relevant parties (e.g. trial participants, ethical committees) {25}

All protocol amendments will require approval from the Sponsor, Research Ethics Committee, Health Research Authority, and site Research and Development departments prior to implementation. Where appropriate, study participants will be notified of protocol amendments, and the requirement for this communication will be determined by the Sponsor and Research Ethics Committee.

### Dissemination plans {31a}

The study findings will be distributed widely through national and international conferences, journal publications, PPIE newsletters and forums.

## Discussion

Individuals requiring maintenance in-centre haemodialysis have poor HRQoL, high symptom burden and adverse clinical outcomes. This is compounded by the restrictive scheduling of in-centre haemodialysis regimens, whereby the individual essentially loses 3 days of life participation every week. In-centre daytime haemodialysis session length is limited to around 4 h, thrice-weekly. This is a protocolised approach to patient care as 12 h of haemodialysis a week generally provides enough clearance to maintain life whilst suiting dialysis unit scheduling and capacity requirements [12]. Thus, the regimen is at the convenience of the service provider and not the service user. Alternative KRT modalities, such as transplantation and home dialysis, are not accessible to all, and there are significant barriers to their uptake. There is increasing observational evidence that extended-hours INHD haemodialysis may have a number of benefits, including quality of life. However, there is a lack of adequately powered RCT evidence specifically assessing the impact of thrice-weekly extended-hours (6 to 8 h per session) INHD on HRQoL in comparison to standard care (in-centre daytime haemodialysis) in a UK population.

The NightLife study will address this clinically important and patient prioritised research question, being the first UK multicentre RCT to compare 6 months of INHD to standard care on HRQoL, clinical and cost-effectiveness and safety. In addition, the NightLife study will expand our understanding of alternative KRT modality adoption and implementation within the NHS, and the recruitment practices and challenges of maintaining clinical equipoise in dialysis research. Thus, the results of the NightLife study will go beyond determining the impact of INHD, but will generate findings and hypotheses for further research applicable to NHS service delivery, implementation science and dialysis RCT design.

## Trial status

The NightLife study commenced on 01/01/2020. The overall study end date is 31/12/2024. The current protocol is version 4.0, 9th February 2023.

## Trial registration

Trial registration number: ISRCTN87042063.

## Data Availability

The NightLife study dataset will be held and analysed by the Leicester Clinical Trials Unit.

## Abbreviations

KRT: Kidney Replacement Therapy
ESKD: End-stage Kidney Disease
HRQoL: Health-Related Quality of Life
INHD: In-centre Nocturnal Haemodialysis
RCT: Randomised clinical trial
FHN: Frequent Haemodialysis Network
QRI: QuinteT Recruitment Intervention
NHS: National Health Service
KDQoL: Kidney Disease Quality of Life instrument
KDQoL-SF: Kidney Disease Quality of Life instrument short form
PCS: Physical Component Summary score
MCS: Mental Component Summary score
KSS: Kidney Summary Score
KDCS: Kidney Disease Component Summary score
PSQI: Pittsburgh Sleep Quality Index
MoCA: Montreal Cognitive Assessment
SONG-HD: Standardised Outcomes in Nephrology Haemodialysis
CACE: Complier Average Causal Effect
SAE: Serious adverse events
MedDRA: Medical Dictionary for Regulatory Activities
QALYs: Quality-adjusted life years
PPIE: Patient Public Involvement and Engagement
ISRCTN: International Standard Randomised Controlled Trials Number
TMG: Trial Management Group
CONSORT: Consolidated Standards of Reporting Trials
ICH-GCP: International Council for Harmonisation of Technical Requirements for Pharmaceuticals for Human Use (ICH) Guideline for Good Clinical Practice (GCP)
GDPR: General Data Protection Regulation
EU: European Union
UKRR: UK Renal Registry
SRR: Scottish Renal Registry
HES: Hospital Episode Statistics
ONS: Office for National Statistics
ISD: Information Services Division

## References

[1] UK Renal Registry. UK renal registry 24th annual report—data to 31/12/2020, Bristol, UK. 2022. Available from https://ukkidney.org/audit-research/annual-report.

[2] Bello AK, Okpechi IG, Osman MA, Cho Y, Htay H, Jha V (2022). Epidemiology of haemodialysis outcomes. Nat Rev Nephrol.

[3] Mapes DL, Bragg-Gresham JL, Bommer J, Fukuhara S, McKevitt P, Wikström B (2004). Health-related quality of life in the Dialysis Outcomes and Practice Patterns Study (DOPPS). Am J Kidney Dis.

[4] Perl J, Karaboyas A, Morgenstern H, Sen A, Rayner HC, Vanholder RC (2017). Association between changes in quality of life and mortality in hemodialysis patients: results from the DOPPS. Nephrol Dial Transplant.

[5] Tentori F, Mapes DL (2010). Opinion: Health-Related Quality of Life and Depression among Participants in the DOPPS: predictors and associations with clinical outcomes. Semin Dial.

[6] Brown EA, Zhao J, McCullough K, Fuller DS, Figueiredo AE, Bieber B (2021). Burden of kidney disease, health-related quality of life, and employment among patients receiving peritoneal dialysis and in-center hemodialysis: findings from the DOPPS program. Am J Kidney Dis..

[7] Valderrábano F, Jofre R, López-Gómez JM (2001). Quality of life in end-stage renal disease patients. Am J Kidney Dis.

[8] Hall RK, Cary MP, Washington TR, Colón-Emeric CS (2020). Quality of life in older adults receiving hemodialysis: a qualitative study. Qual Life Res.

[9] Kierans CM, Maynooth N (2001). Sensory and narrative identity: the narration of illness process among chronic renal sufferers in Ireland. Anthropol Med.

[10] de Jong RW, Boezeman EJ, Chesnaye NC, Bemelman FJ, Massy ZA, Jager KJ (2022). Work status and work ability of patients receiving kidney replacement therapy: Results from a European survey. Nephrol Dial Transplant.

[11] Kerr M, Bray B, Medcalf J, O'Donoghue DJ, Matthews B (2012). Estimating the financial cost of chronic kidney disease to the NHS in England. Nephrol Dial Transplant..

[12] Ashby D, Borman N, Burton J, Corbett R, Davenport A, Farrington K (2019). Renal association clinical practice guideline on haemodialysis. BMC Nephrol.

[13] Oberley ET, Schatell DR (1996). Home hemodialysis: survival, quality of life, and rehabilitation. Adv Ren Replace Ther.

[14] Miller BW, Himmele R, Sawin D-A, Kim J, Kossmann RJ (2018). Choosing home hemodialysis: a critical review of patient outcomes. Blood Purif.

[15] Young BA, Chan C, Blagg C, Lockridge R, Golper T, Finkelstein F (2012). How to overcome barriers and establish a successful home HD program. Clin J Am Soc Nephrol.

[16] Chan CT, Wallace E, Golper TA, Rosner MH, Seshasai RK, Glickman JD (2019). Exploring barriers and potential solutions in home dialysis: an NKF-KDOQI conference outcomes report. Am J Kidney Dis.

[17] Gong Y, Xie L, Yu S (2022). Long-term in-center nocturnal hemodialysis improves renal anemia and malnutrition and life quality of older patients with chronic renal failure. Clin Interv Aging..

[18] Dumaine CS, Ravani P, Parmar MK, Leung KC, MacRae JM (2022). In-center nocturnal hemodialysis improves health-related quality of life for patients with end-stage renal disease. J Nephrol.

[19] Bugeja A, Dacouris N, Thomas A, Marticorena R, McFarlane P, Donnelly S (2009). In-center nocturnal hemodialysis: another option in the management of chronic kidney disease. Clin J Am Soc Nephrol.

[20] Graham-Brown MP, Churchward DR, Smith AC, Baines RJ, Burton JO (2015). A 4-month programme of in-centre nocturnal haemodialysis was associated with improvements in patient outcomes. Clin Kidney J.

[21] Lacson E, Xu J, Suri RS, Nesrallah G, Lindsay R, Garg AX (2012). Survival with three-times weekly in-center nocturnal versus conventional hemodialysis. J Am Soc Nephrol.

[22] David S, Kümpers P, Eisenbach GM, Haller H, Kielstein JT (2009). Prospective evaluation of an in-centre conversion from conventional haemodialysis to an intensified nocturnal strategy. Nephrol Dial Transplant.

[23] Graham-Brown MP, Churchward DR, Hull KL, Preston R, Pickering WP, Eborall HC (2017). Cardiac remodelling in patients undergoing in-centre nocturnal haemodialysis: results from the MIDNIGHT study, a non-randomized controlled trial. Blood Purif.

[24] Ok E, Duman S, Asci G, Tumuklu M, Onen Sertoz O, Kayikcioglu M (2011). Comparison of 4-and 8-h dialysis sessions in thrice-weekly in-centre haemodialysis: a prospective, case-controlled study. Nephrol Dial Transplant.

[25] Burton JO, Graham-Brown MP (2018). Nocturnal hemodialysis: an underutilized modality?. Curr Opin Nephrol Hypertens.

[26] Hull KL, Quann N, Glover S, Wimbury C, Churchward DR, Pickering WP (2021). Evaluating the clinical experience of a regional in-center nocturnal hemodialysis program: The patient and staff perspective. Hemodial Int.

[27] Culleton BF, Walsh M, Klarenbach SW, Mortis G, Scott-Douglas N, Quinn RR (2007). Effect of frequent nocturnal hemodialysis vs conventional hemodialysis on left ventricular mass and quality of life: a randomized controlled trial. JAMA.

[28] Rocco MV, Lockridge RS, Beck GJ, Eggers PW, Gassman JJ, Greene T (2011). The effects of frequent nocturnal home hemodialysis: the Frequent Hemodialysis Network Nocturnal Trial. Kidney Int.

[29] Jardine MJ, Zuo L, Gray NA, De Zoysa JR, Chan CT, Gallagher MP (2017). A trial of extending hemodialysis hours and quality of life. J Am Soc Nephrol.

[30] Daugirdas JT, Greene T, Rocco MV, Kaysen GA, Depner TA, Levin NW (2013). Effect of frequent hemodialysis on residual kidney function. Kidney Int.

[31] Hull KL, March DS, Churchward DR, Graham-Brown MP, Burton JO (2020). The effect of extended-hours hemodialysis on outcomes: A systematic review and meta-analysis. Hemodial Int.

[32] Donovan JL, Rooshenas L, Jepson M, Elliott D, Wade J, Avery K (2016). Optimising recruitment and informed consent in randomised controlled trials: the development and implementation of the Quintet Recruitment Intervention (QRI). Trials.

[33] Peipert JD, Bentler PM, Klicko K, Hays RD (2018). Psychometric properties of the kidney disease quality of life 36-item short-form survey (KDQOL-36) in the United States. Am J Kidney Dis.

[34] Hays RD, Kallich J, Mapes D, Coons S, Amin N, Carter W (1997). Kidney disease quality of life short form (KDQOL-SF™), version 1.3: a manual for use and scoring.

[35] Mapes DL, Lopes AA, Satayathum S, McCullough KP, Goodkin DA, Locatelli F (2003). Health-related quality of life as a predictor of mortality and hospitalization: the Dialysis Outcomes and Practice Patterns Study (DOPPS). Kidney Int.

[36] SONG-HD. Standardised Outcomes in Nephrology: SONG-HD 2015 [Available from: https://songinitiative.org/projects/song-hd/. Accessed 19 April 2023.

[37] Macdougall IC, White C, Anker SD, Bhandari S, Farrington K, Kalra PA (2019). Intravenous iron in patients undergoing maintenance hemodialysis. N Engl J Med.

[38] Adamson J, Cockayne S, Puffer S, Torgerson DJ (2006). Review of randomised trials using the post-randomised consent (Zelen's) design. Contemp Clin Trials.

[39] Cook TD, Campbell DT, Day A. Quasi-experimentation: design & analysis issues for field settings. Boston: Houghton Mifflin Boston; 1979.

[40] Onghena P. Resentful demoralization. Encyclopedia of statistics in behavioral science. Chichester: Wiley; 2005.

[41] Rooshenas L, Elliott D, Wade J, Jepson M, Paramasivan S, Strong S (2016). Conveying equipoise during recruitment for clinical trials: qualitative synthesis of clinicians’ practices across six randomised controlled trials. PLoS Med.

[42] Rooshenas L, Paramasivan S, Jepson M, Donovan JL (2019). Intensive triangulation of qualitative research and quantitative data to improve recruitment to randomized trials: the quintet approach. Qual Health Res.

[43] Sealed Envelope. Randomisation and online databases for clinical trials. 2001 [Available from: https://www.sealedenvelope.com/. Accessed May 3 2023.

[44] Hernán MA, Robins JM (2017). Per-protocol analyses of pragmatic trials. N Engl J Med.

[45] Cluley V (2017). Using photovoice to include people with profound and multiple learning disabilities in inclusive research. Br J Learn Disabil.

[46] Jones KC, Burns A. Unit costs of health and social care 2021 2021 [Available from: https://www.pssru.ac.uk/project-pages/unit-costs/unit-costs-of-health-and-social-care-2021/.

[47] NICE. British National Formulary (BNF) 2022 [Available from: https://bnf.nice.org.uk/. Accessed January 23 2023.

[48] NHS. NHS Reference Costs 2020/21 [Available from: https://www.england.nhs.uk/costing-in-the-nhs/national-cost-collection/#ncc1819. Accessed January 23 2023.

[49] Siebert U, Alagoz O, Bayoumi AM, Jahn B, Owens DK, Cohen DJ (2012). State-transition modeling: a report of the ISPOR-SMDM modeling good research practices task force-3. Value in Health.

[50] United States Renal Data System. 2022 USRDS Annual Data Report: Epidemiology of kidney disease in the United States. Bethesda: National Institutes of Health, National Institute of Diabetes and Digestive and Kidney Diseases; 2022. https://usrds-adr.niddk.nih.gov/2022/suggested-citation#:~:text=Suggested%20citation%20for%20this%20report,%2C%20Bethesda%2C%20MD%2C%202022.

[51] Fotheringham J, Barnes T, Dunn L, Lee S, Ariss S, Young T (2021). A breakthrough series collaborative to increase patient participation with hemodialysis tasks: a stepped wedge cluster randomised controlled trial. PLoS ONE.

[52] MedDRA. Medical Dictionary for Regulatory Activities [Available from: https://www.meddra.org/. Accessed May 3 2023.

